# Vaping, smoking and risk of early onset lung cancer

**DOI:** 10.3389/fonc.2025.1741978

**Published:** 2026-01-06

**Authors:** Marisa A. Bittoni, David P. Carbone, Randall E. Harris

**Affiliations:** Colleges of Medicine and Public Health, and Comprehensive Cancer Center, The Ohio State University, Columbus, OH, United States

**Keywords:** adenocarcinoma, cigarette smoking, electronic cigarettes, lung cancer, vaping

## Abstract

Lung cancer is the leading cause of cancer mortality worldwide, including young adults. While it is well known that cigarette smoking is the dominant risk factor for lung cancer, the inhalation of heated aerosolized vaping oil has now replaced cigarette smoking as the major source of nicotine among young people. We, therefore, studied the potential role of both vaping and cigarette smoking in the development of early onset lung cancer. Using a case-control study design, we compared the smoking and vaping habits of 256 young adults (< 50 years of age) diagnosed with pathologically confirmed lung cancer to that of 2,921 control subjects without cancer that were group matched by age (within 5 years), sex, race and location to the cases. The odds ratio for those who both vaped and smoked (OR = 13.8, 95% CI: 7.7-24,9) was 2.8 times higher than for smoking alone (OR = 5.0, 95% CI: 3.7-6.9). Pulmonary adenocarcinomas accounted for 72% of the early onset lung tumors among the cases, and risk estimates for this specific cell type were 3.7 times higher for those who smoked and vaped (OR = 14.8, 95% CI = 8.0-27.4) compared to those who only smoked (OR = 4.0, 95% CI = 2.9-5.6). Our findings suggest that compared to smoking alone, vaping and smoking together accelerate lung cancer risk among young people, particularly in the development of pulmonary adenocarcinoma.

## Introduction

Lung cancer is the leading cause of cancer mortality worldwide (1). While the age-adjusted incidence and mortality rates of lung cancer across all ages have decreased in the 21^st^ century (2), the rates have actually *increased* among young adults in Asian populations, particularly among young women (2). Recent studies also consistently show that young women have higher incidence rates of lung cancer than young men, a pattern that is not fully explained by sex differences in cigarette smoking (3, 4).

The dominant risk factor for lung cancer is chronic cigarette smoking, which is estimated to cause 87% of all lung cancer deaths in the United States (5). Nevertheless, prevalence rates of cigarette smoking have declined in many populations and other sources of nicotine delivery, particularly the use of e-cigarettes (vaping) have now replaced smoking. Notably, the inhalation of heated aerosolized vaping oil is currently the major source of nicotine among young people. During 2024, over 1.6 million middle and high school students in the United States reported current electronic cigarette use, thus making vaping the most widely used tobacco product among youth (6). These products are aggressively marketed with appealing taste, design and flavors, all designed to increase the frequency of use and dependence. Adolescents are especially vulnerable to nicotine addiction due to ongoing brain development, which may result in susceptibility to adverse long-term health consequences (7, 8).

A 2024 random-effects meta-analysis of 107 cross-sectional and longitudinal studies reported that current nicotine vaping, compared with no nicotine use, was associated with increased rates of asthma, chronic obstructive pulmonary disease (COPD), periodontitis, cardiovascular disease, and stroke (pooled odds ratios of 1.24-1.47) (9). A single vaping session can cause increased bodily inflammation, with one study reporting a 30% increase in oxidative stress as measured by CD45+ cell markers (10).

Aerosols from electronic cigarettes have also been found to contain a mixture of carcinogens, including aldehydes (e.g., formaldehyde, acetaldehyde), volatile organic compounds, and heavy metals. These compounds are known to cause DNA damage, oxidative stress, and chronic inflammation—hallmarks of carcinogenesis. A 2023 study found that exclusive vapers exhibited levels of DNA damage in oral epithelial cells comparable to smokers, with damage increasing based on frequency of use and flavor type (11, 12).

While the inhalation of aerosolized nicotine-containing vaping oil is known to expose the lungs to carcinogens, only a few studies have examined the potential role of vaping as a lung cancer risk factor. Rather, much attention has focused on the lower concentrations of carcinogens in vape aerosol compared to cigarette smoke, and the potential for “harm reduction” with substitution of vaping for smoking (13–18).

Counter to the concept that substituting vaping for smoking promotes ‘harm reduction’, Tang and colleagues recently published an animal study revealing that exposure to aerosolized vape oil containing nicotine resulted in the development of pulmonary adenocarcinoma and bladder dysplasia in mice (19). Notably, molecular studies of the tumors from these mice showed the presence of a ‘*signature pattern of mutations*’ distinctly different from that found in human lung cancer and consistent with base pair changes arising from exposure to carbonyl aldehydes (20).

A few recent studies of human lung cancer have also noted significant increases in the risk of lung cancer associated with vaping. In a large case control study, Bittoni and colleagues (21) observed that vaping combined with chronic smoking elevated the risk of lung cancer four times higher than chronic cigarette smoking alone. This dual-use pattern was eight times higher among lung cancer patients than controls, highlighting the additive risk of vaping in combination with smoking. Other studies have also observed that vaping increases the risk of lung cancer development (22, 23). Additionally, a systematic review published in 2025 concluded that vaping exposure is consistently associated with biomarkers of genotoxicity, oxidative stress, and tumor growth (24).

In view of these recent findings, it is essential to continue the investigation of vaping in the development of lung cancer, particularly in young people who initiated the vaping habit at an early age. No prior study has specifically evaluated vaping and smoking in early onset lung cancer. In the current report, we present the results of a case control study of vaping, cigarette smoking and early onset lung cancer.

## Methods

**Figure 1:**
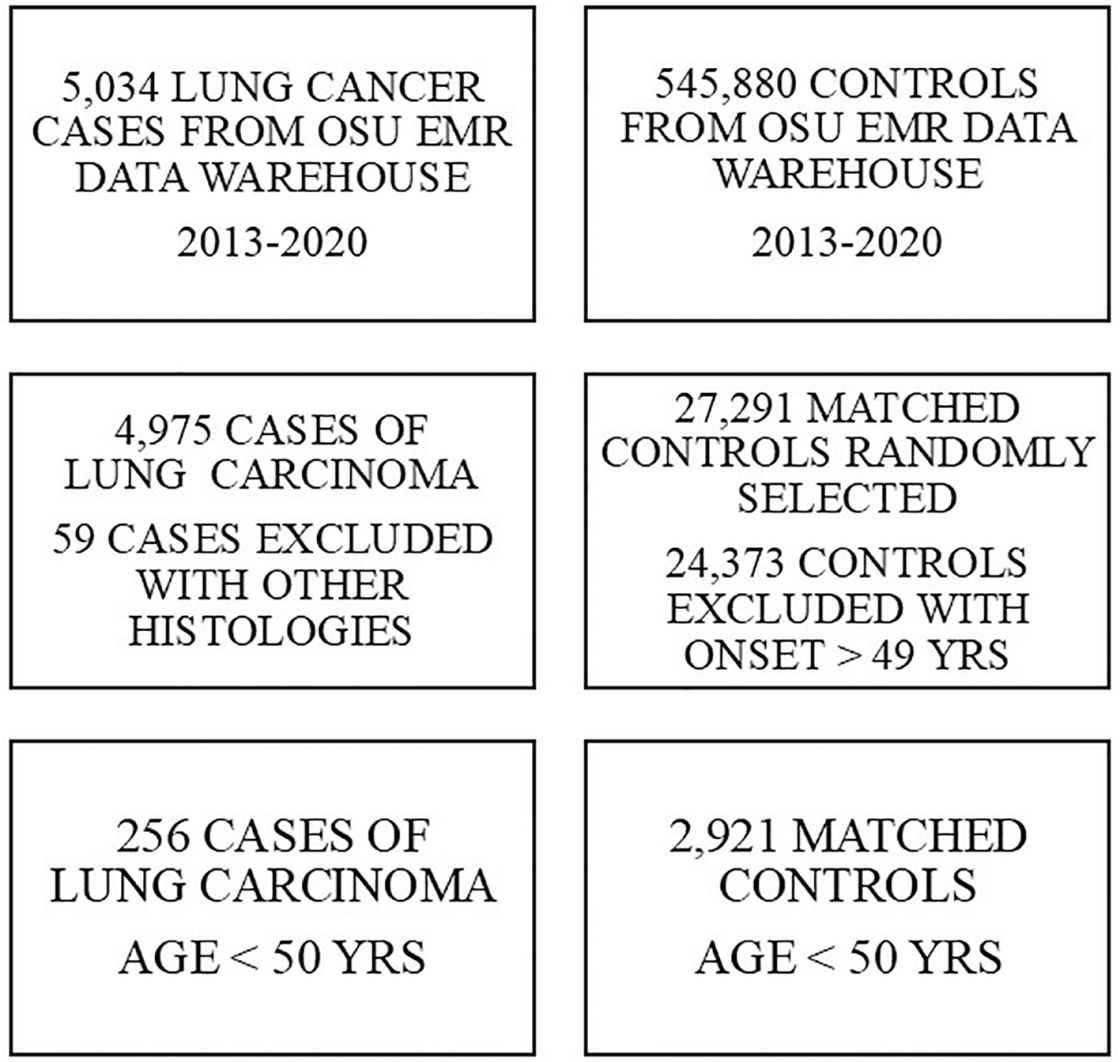
Flow diagram of sample ascertainment. *Cases of pathologically confirmed lung cancer and matched controls without cancer were ascertained from the Electronic Medical Records (EMR) Data Warehouse maintained by The Ohio State University Medical Center. Controls were group matched to the cases by age (five year age categories), gender, race, county of residence and year of ascertainment.

We conducted a case-control study to examine the potential role of vaping as a risk factor for lung cancer in young adults. Using the repository of electronic medical records of The Ohio State University Medical Center, we ascertained data on cigarette smoking, use of electronic cigarettes (vaping) and other potential risk factors for 256 young adults (less than 50 years of age) diagnosed with pathologically confirmed carcinoma of the lung during the time period 2013-2021. We also ascertained data for 2,921 control subjects without cancer who visited OSU clinics for routine checkups that were group-matched at a 10:1 ratio to the cases by age, sex, race, location of residence and time (year) of patient visit. A flow diagram of the ascertainment of samples of cases and controls included for study is shown in **Figure 1**. All control subjects were accessed through outpatient clinics for routine annual checkups. Variables of interest included reported comorbid conditions (coronary artery disease, chronic obstructive pulmonary disease), cigarette smoking, and use of e-cigarettes (vaping). Daily current cigarette smoking and vaping were self-reported at the time of patient intake. No personal health information was retrieved in the study, thereby retaining anonymity of all study participants, and the study protocol was approved by the Human Subjects Review Board of The Ohio State University Medical Center (Protocol Number 2019C0105).

Odds ratios were estimated by logistic regression analysis for vaping and cigarette smoking with adjustment for matching variables (age, sex, gender) as well as comorbid conditions, chronic obstructive pulmonary disease (COPD) and coronary artery disease (CAD). Separate estimates were computed for men and women, and for distinct histologic cell types, such as pulmonary adenocarcinoma. Prevalent comorbid conditions, including coronary artery disease (CAD) and COPD, were also included in regression models to test for effect modification. Based on our sample size of 256 cases and 2,291 controls with type 1 error set at 0.05 and assuming a prevalence of 5% of vaping among smokers, the study had 80% power to detect an odds ratio of 2 for vaping and smoking (dual use) compared to smoking alone.

## Results

**Table 1** shows demographic and selected clinical characteristics of the enrolled subjects by case-control status. Matching resulted in close agreement in the distributions of age, sex and race between the lung cancer cases and control subjects. Cases had significantly higher prevalence rates of COPD and CAD (63.3% and 33.4%) than controls (18.0% and 12.3%, P<0.01).

**Table 1:** Characteristics of lung cancer cases and control subjects.

Variable	Cases (256)	Controls (2921)
	n (%)	n (%)
Age at Diagnosis		
18-29	6 (1.6)	106 (3.6)
30-39	34 (14.0)	438 (15.0)
40-49	216 (84.4)	2377 (81.4)
Gender		
Women	131 (51.2)	1521 (52.1)
Men	125 (48.8)	1400 (47.9)
Race		
White	211 (82.4)	2361 (80.9)
Black	40 (15.6)	531 (18.2)
Other	5 (2.0)	29 (1.0)
Comorbidities*		
COPD	162 (63.3)	528 (18.0)
CAD	87 (34.0)	367 (12.6)

*COPD, Chronic Obstructive Pulmonary Disease; CAD, Coronary Artery Disease.

**Table 2** shows the exposure data and odds ratios (OR) for smoking combined with vaping compared to smoking alone. Smoking and vaping among cases was over five times higher than among controls (7.8% versus 1.5%, P<0.01) and the OR for those who both vaped and smoked (OR = 13.8, 95% CI: 7.7-24,9) was almost 2.8 times higher than for smoking alone (OR = 5.0, 95% CI: 3.7-6.9, P<0.01).

**Table 2:** Odds ratios for vaping and cigarette smoking in lung cancer cases and controls*.

Status	Cases (256) n (%)	Controls (2921) n (%)	OR (95% CI)
Vapers & Smokers	20 (7.8)	45 (1.5)	13.8 (7.7-24.9)
Smokers Only	179 (69.9)	1105 (37.8)	5.0 (3.7-6.9)
Nonsmokers	57 (22.3)	1771 (60.7)	1.0

*ORs are adjusted for matched variables of age, gender and race.

**Table 3** shows lung cancer OR estimates for comorbid conditions, COPD and CAD, as well as estimates for smoking and vaping and smoking alone with additional adjustment for these comorbidities. The OR for COPD and CAD were both elevated (4.9 and 3.2), but adjustment for these comorbidities did not diminish the significantly higher risk (P<0.05) for smokers and vapers compared to smokers.

**Table 3:** Odds ratios for vaping and cigarette smoking in lung cancer cases and controls by comorbidities*.

Comorbidity	Condition	Vapers/Smokers	Smokers
COPD	4.9 (3.6-6.5)	8.1 (4.3-15.2)	3.0 (2.2-4.2)
CAD	3.2 (2.4-4.3)	14.0 (7.7-25.4)	4.7 (3.4-6.4)

*ORs are adjusted for matched variables of age, gender, race and comorbidities (COPD and CAD).

**Table 4** shows separate results for males and females and for pulmonary adenocarcinoma, the dominant histologic cell type (72%) among the lung cancer cases. Results reflect higher risk estimates for males than females but the significantly higher risk for smoking combined with vaping compared to smoking alone was observed for all genders. Notably, the risk estimates for pulmonary adenocarcinoma reflect 3.7 times higher risk for smoking combined with vaping compared to smoking alone (14.8 versus 4.0, P<0.01).

**Table 4:** Distribution by gender and histologic cell type for lung cancer cases with corresponding odds ratios*.

Variable	Cases	Vapers/Smokers	Smokers
	n (%)	OR (95% CI)	OR (95% CI)
Gender			
Male	125 (48.8)	24.6 (10.3-58.8)	7.1 (4.3-11.5)
Female	131 (51.2)	9.5 (4.1-22.0)	3.8 (2.5-5.7)
Histology			
Adenocarcinoma	185 (72.3)	14.8 (8.0-27.4)	4.0 (2.9-5.6)

*ORs are adjusted for matched variables of age, gender and race.

**Figure 2:**
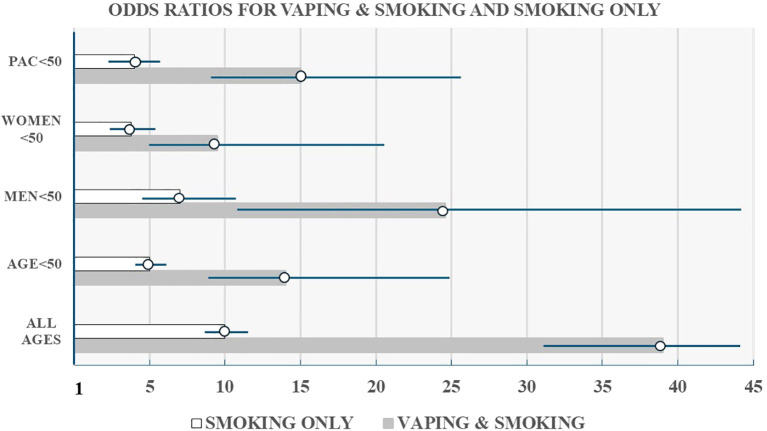
Odds ratios with 95% confidence intervals for chronic smokers who vaped versus chronic smokers who did not vape by age, gender, and histologic cell type (pulmonary adenocarcinoma).

**Figure 2** provides a visual comparison of the risk estimates for smoking combined with vaping compared to smoking alone for individuals of all ages from our previous investigation (21) compared to those under age 50 with separate estimates for men, women and pulmonary adenocarcinoma. While the risk estimates for the younger cases are lower than for all ages, it is notable that our findings for early onset lung cancer continued to reflect 3-4-fold higher risk for dual users compared to smoking alone.

## Discussion

Our findings provide the first evidence that smoking in combination with vaping significantly increases the risk of lung cancer in adults under age 50. Risk estimates reflect a nearly three-fold higher risk of lung cancer among those under age 50 who combined vaping and smoking cigarettes (OR = 13.8) compared to those who only smoked (OR = 5.0). The dominant histological cell type in our study was pulmonary adenocarcinoma (present in 72% of cases) and separate risk estimates for this cell type showed an even higher risk differential (3.7) for smoking and vaping (OR = 14.8) compared to smoking alone (OR = 4.0).

The current results for early onset lung cancer corroborate our previous work showing a 4-fold increased risk of lung cancer in a large sample of 4,975 lung cancer cases of all ages (21). These findings suggest that the addition of vaping to smoking has a potentially synergistic effect and support the premise that pyrolysis of vaping oil containing nicotine produces harmful substances that accelerate carcinogenesis. Biomarker studies have shown that youth who vape are exposed to elevated levels of multiple carcinogens including formaldehyde, acetaldehyde, acrylamide, toluene, and tobacco-specific nitrosamines (24, 25). Recent research has also identified shared DNA methylation changes in vapers and smokers, including epigenetic alterations in tumor suppressor genes such as HIC1, which are linked to lung cancer development (12). Aside from toxic chemicals in aerosolized vaping oil, heating elements in electronic cigarettes use may also potentially expose users to heavy metals from batteries and heating coils that may be carcinogenic or toxic to the heart and lungs (26, 27). These findings raise concerns about vaping as a contributing factor to lung cancer pathogenesis beyond traditional cigarette smoking-related mechanisms.

Our results are in marked contrast to the “harm reduction” approach that deems vaping to be less harmful than cigarette smoking, instead showing that exposure to aerosolized e-liquid may in fact promote lung carcinogenesis, especially when combined with smoking. The elevated risks observed across demographic and clinical subgroups underscore the need for targeted public health interventions and regulatory policies aimed at reducing dual use among youth and high-risk populations. One study limitation was that due to the nature of the electronic medical record data, we could not quantify vaping and smoking as detailed as we had planned, nor the timing of vaping relative to smoking. Nevertheless, exposure estimates for controls do not reflect evidence of selection bias or “healthy user bias”, e.g., among our sample of 2,291 healthy controls, the overall prevalence of vaping and smoking was 1.5% and the prevalence of vaping among smokers was 3.9%, consistent with concurrent estimates for the general USA population, 1.3% and 3.9%, respectively (28, 29). Another limitation was the lack of information on potential effect modifiers such as exposure to secondhand smoke, air pollution, and genetic predisposition to lung cancer. Clearly, future well-designed studies focused on adolescents and young adults are urgently needed to clarify the long-term impact of vaping on lung cancer risk and to inform regulatory and prevention strategies.

## Conclusion

Dual use of vaping and smoking is associated with significantly increased risk of lung cancer among young adults, particularly in the development of pulmonary adenocarcinoma. Findings highlight the urgent need for longitudinal research and policy efforts to address the growing prevalence of vaping, especially among youth and chronic smokers.

## Data Availability

The raw data supporting the conclusions of this article will be made available by the authors, without undue reservation.
